# Supplementary material for: Urine 5-Hydroxyindoleacetic Acid Negatively Correlates with Migraine Occurrence and Characteristics in the Interictal Phase of Episodic Migraine
Source: Int J Mol Sci. 2024 May 17;25(10):5471. doi: 10.3390/ijms25105471 (PMC11121987; doi:10.3390/ijms25105471)
Supplement: Supplementary file 1 [file ijms-25-05471-s001.zip › ijms-2978766-supplementary.pdf]

Table S1. Age (years) of the individuals of the control group and migraine patients.

No.	Control	Migraine
1	36	26
2	41	42
3	39	26
4	29	28
5	36	26
6	45	48
7	42	56
8	41	26
9	24	32
10	33	65
11	51	26
12	36	49
13	27	26
14	39	45
15	28	41
16	44	26
17	29	48
18	51	38
19	42	
20	37	
21	33	
22	48	
23	49	
24	56	
25	49	
26	35	
27	38	
28	48	
29	28	
30	32	
31	41	
32	47	
Mean	39	39
Median	39	41
Range	24-56	26-65
Standard deviation	8	12
p (Mann-Whitney)	0.910	

Table S2. Sex of the individuals of the control group and migraine patients.

No.	Control	Migraine
1	M[1)]	F
2	M	F
3	F	F
4	F	M
5	F	M
6	M	F
7	F	F
8	M	F
9	F	M
10	M	F
11	M	F
12	M	F
13	M	F
14	M	F
15	F	F
16	M	M
17	F	F
18	F	M
19	M	M
20	F	F
21	F	F
22	F	
23	M	
24	F	
25	F	
26	F	
27	M	
28	M	
29	F	
30	F	
31	F	
32	M	
p (Mann-Whitney)	0.254	

[1)] M – male, F – female

Table S3. Characteristics of the migraine patients in the categories of the severity of migraine evaluated by Migraine Disability Assessment Scale (MIDAS) score, monthly migraine days (MMD), monthly headache days (MHD) and the Patient Health Questionnaire 9 (PHQ-9).

No.	MIDAS	MMD	MHD	PHQ-9
1	16	4	8	5
2	26	10	14	14
3	14	4	6	6
4	8	2	4	4
5	12	4	6	9
6	18	6	8	7
7	10	3	5	8
8	9	2	4	4
9	20	8	10	6
10	5	1	3	10
11	15	4	6	5
12	7	2	3	4
13	8	2	4	9
14	11	3	5	11
15	4	1	2	3
16	3	1	1	2
17	5	2	3	4
18	4	1	2	3
19	3	1	1	1
20	7	2	3	4
21	7	4	6	6

Table S4. Tryptophan (TRP) and its main metabolites in migraine patients and controls. Urinary levels of tryptophan (TRP), 5-hydroxyaminoacetic acid (5-HIAA), kynurenine (KYN), kynurenic acid (KYNA), and quinolinic acid (QA) were expressed in milligrams per gram of creatinine (mg/gCr).

No.	TRP		5-HIAA		KYN		KYNA		QA	
	Control	Migraine	Control	Migraine	Control	Migraine	Control	Migraine	Control	Migraine
1	12.8	19.48	3.2	1.79	0.5	1.08	3.16	2.07	2.65	7.03
2	10.6	17.69	3.6	2.99	0.5	0.38	1.92	1.34	2.41	7.49
3	14.6	6.23	2.3	1.17	0.4	0.28	2.46	0.89	4.25	5.75
4	16.2	8.09	3.1	4.06	0.3	0.24	2.53	2.4	3.28	3.27
5	14.2	13.39	2.3	0.5	0.5	0.5	1.62	2.66	1.24	9.67
6	15.1	15.33	2.7	0.98	0.2	0.85	1.68	2.28	3.26	5.58
7	9.4	12.93	4.4	0.99	0.5	0.47	1.46	1.98	4.24	0.87
8	15.1	8.59	3.8	1.81	0.4	0.21	2.54	0.77	5.12	3.6
9	14.6	10.58	3.6	1.48	0.5	0.69	2.94	2.18	3.16	3.36
10	14.2	13.23	3.7	3.08	0.8	0.65	1.68	2.29	2.72	4.27
11	12.6	17.31	4.8	2.31	0.4	1.17	2.73	2.5	1.92	8.81
12	15.8	17.17	3.1	4.05	0.6	1.37	2.16	3.39	2.14	4.63
13	13.4	6.08	2.4	3.19	0.5	0.55	2.62	1.42	3.06	6.13
14	14.6	10.22	4.6	2.87	0.3	0.43	1.93	1.35	4.53	7.24
15	12.1	9.35	3.8	2.32	0.5	0.41	2.48	1.74	3.12	8.18
16	16.2	9.28	1.7	4.03	0.3	0.9	2.12	2.88	1.92	9.16
17	15.5	11.91	2.2	3.16	0.5	0.57	1.46	1.9	2.84	2.9
18	16.4	16.08	3.1	3.06	0.4	1.29	2.43	2.32	3.76	4.87
19	14.3	18.09	4.8	3.02	0.6	0.92	2.84	1.26	4.16	4.53
20	14.4	12.72	4.6	3.28	0.5	1.34	2.21	2.27	1.23	4.15
21	15.6	11.03	4.3	3.01	0.7		2.86		2.38	
22	12.3		3.2		0.3		1.95		2.42	
23	12.6		3.1		0.4		2.65		3.58	
24	11.5		1.3		0.4		2.98		4.12	
25	12.9		3.6		0.5		3.16		3.23	
26	12.6		3.1		0.4		2.36		4.85	
27	15.3		4.2		0.5		3.16		3.12	
28	14.2		2.6		0.4		2.78		2.65	
29	13.2		3.8		0.5		2.74		3.33	
30	12.5		3.4		0.4		2.44		1.86	
31	11.2		3.2		0.5		1.82		4.16	
32	14.6		2.3		0.4		2.38		3.32	
$p^{2)}$	0.241		0.008		0.015		0.021		< 0.001	

$^{2)}$ Mann-Whitney test

Table S5. Ratios of urinary levels of 5-hydroxyaminoacetic acid (5-HIAA) and kynurenine (KYN) to tryptophan (TRP), KYN to kynurenic acid (KYNA), and KYNA to quinolinic acid (QA) in migraine patients and controls.

No.	5-HIAA/TRP		KYN/TRP		KYNA/KYN		KYNA/QA	
	Control	Migraine	Control	Migraine	Control	Migraine	Control	Migraine
1	0.25	0.06884	0.04	0.06	6.32	1.92	1.19	0.29
2	0.33	0.15349	0.05	0.02	3.84	3.53	0.79	0.18
3	0.16	0.06613	0.03	0.05	6.15	3.18	0.55	0.15
4	0.19	0.65168	0.02	0.03	8.433	3.23	0.77	0.73
5	0.16	0.06180	0.05	0.12	3.24	5.32	1.3	0.28
6	0.17	0.07318	0.02	0.06	8.4	2.68	0.5	0.4
7	0.32	0.06457	0.05	0.04	2.92	4.21	0.34	0.28
8	0.25	0.13998	0.03	0.03	6.35	3.67	0.49	0.21
9	0.24	0.17229	0.03	0.07	5.88	6.06	0.93	1.24
10	0.26	0.29111	0.07	0.05	2.1	3.52	0.61	0.54
11	0.38	0.17460	0.03	0.07	6.825	2.14	1.42	0.28
12	0.19	0.23396	0.04	0.08	3.6	3.93	1.1	1.16
13	0.18	0.18578	0.05	0.09	5.24	2.58	0.85	0.23
14	0.31	0.47203	0.02	0.04	6.433	3.14	0.42	0.1
15	0.11	0.22700	0.04	0.04	4.96	4.24	0.76	0.21
16	0.15	0.43101	0.03	0.1	7.066	3.2	1.1	0.31
17	0.14	0.34051	0.04	0.05	2.92	3.33	0.51	0.66
18	0.18	0.25692	0.02	0.21	6.075	0.71	0.64	0.48
19	0.33	0.18781	0.06	0.05	4.733	1.37	0.69	0.28
20	0.31	0.18131	0.03	0.11	4.42	2.44	1.79	0.79
21	0.27	0.23663	0.04	0.09	4.085	1.96	1.24	0.4
22	0.26		0.03		6.5		0.8	
23	0.24		0.03		6.625		0.74	
24	0.11		0.02		7.45		0.72	
25	0.27		0.04		6.32		0.72	
26	0.24		0.03		5.9		0.97	
27	0.27		0.03		6.32		0.48	
28	0.18		0.03		6.95		1.01	
29	0.29		0.04		5.48		0.84	
30	0.27		0.04		6.1		1.31	
31	0.28		0.04		3.64		0.44	
32	0.16		0.03		5.95		0.71	
P[3)]	0.2		< 0.001		< 0.001		< 0.001	

[3)] Mann-Whitney test

Table S6. The severity of migraine evaluated by Migraine Disability Assessment Scale (MIDAS) score and urine concentration of 5- hydroxyaminoacetic acid (5-HIAA) measured in mg per g of creatinine (gCr) and the ratio of 5-HIAA to tryptophan (TRP) in migraine patients.

No.	MIDAS	5-HIAA	5-HIAA/TRP
1	16	1.79	0.23
2	26	2.99	0.17
3	14	1.17	0.19
4	8	4.96	0.61
5	12	0.5	0.04
6	18	0.98	0.06
7	10	0.99	0.08
8	9	1.81	0.21
9	20	1.48	0.14
10	5	4.08	0.3
11	15	2.31	0.13
12	7	4.54	0.26
13	8	4	0.66
14	11	2.87	0.28
15	4	2.32	0.25
16	3	4.48	0.48
17	5	3.16	0.26
18	4	4.06	0.32
19	3	4.06	0.22
20	7	4.48	0.35
21	7	3.37	0.31

Table S7. Monthly migraine days (MMD) and urine concentration of 5- hydroxyaminoacetic acid (5-HIAA) measured in mg per g of creatinine (gCr) and the ratio of 5-HIAA to tryptophan (TRP) in migraine patients.

No.	MMD	5-HIAA	5-HIAA/TRP
1	4	1.79	0.23
2	10	2.99	0.17
3	4	1.17	0.19
4	2	4.96	0.61
5	4	0.5	0.04
6	6	0.98	0.06
7	3	0.99	0.08
8	2	1.81	0.21
9	8	1.48	0.14
10	1	4.08	0.3
11	4	2.31	0.13
12	2	4.54	0.26
13	2	4	0.66
14	3	2.87	0.28
15	1	2.32	0.25
16	1	4.48	0.48
17	2	3.16	0.26
18	1	4.06	0.32
19	1	4.06	0.22
20	2	4.48	0.35
21	4	3.37	0.31

Table S8. Monthly headache days (MHD) and the urine concentration of 5- hydroxyaminoacetic acid (5-HIAA) measured in mg per g of creatinine (gCr) in migraine patients.

No.	MHD	5-HIAA
1	8	1.79
2	14	2.99
3	6	1.17
4	4	4.96
5	6	0.5
6	8	0.98
7	5	0.99
8	4	1.81
9	10	1.48
10	3	4.08
11	6	2.31
12	3	4.54
13	4	4
14	5	2.87
15	2	2.32
16	1	4.48
17	3	3.16
18	2	4.06
19	1	4.06
20	3	4.48
21	6	3.37

Table S9. The Patient Health Questionnaire 9 (PHQ-9) scores and urine concentration of 5- hydroxyaminoacetic acid (5-HIAA) measured in mg per g of creatinine (gCr) in migraine patients.

No.	PHQ-9 score	5-HIAA
1	5	1.79
2	14	2.99
3	6	1.17
4	4	4.96
5	9	0.5
6	7	0.98
7	8	0.99
8	4	1.81
9	6	1.48
10	10	4.08
11	5	2.31
12	4	4.54
13	9	4
14	11	2.87
15	3	2.32
16	2	4.48
17	4	3.16
18	3	4.06
19	1	4.06
20	4	4.48
21	6	3.37
